# Supplementary material for: FiCoS: A fine-grained and coarse-grained GPU-powered deterministic simulator for biochemical networks
Source: PLoS Comput Biol. 2021 Sep 9;17(9):e1009410. doi: 10.1371/journal.pcbi.1009410 (PMC8476010; doi:10.1371/journal.pcbi.1009410)
Supplement: S2 Text — (PDF) [file pcbi.1009410.s002.pdf]

FiCoS: a fine-grained and coarse-grained GPU-powered deterministic simulator for biochemical networks

Andrea Tangherloni, Marco S. Nobile, Paolo Cazzaniga, Giulia Capitoli,
Simone Spolaor, Leonardo Rundo, Giancarlo Mauri, and Daniela Besozzi

Input files

To correctly perform a simulation of a reaction-based model (RBM), FiCoS requires a list of input files, as reported in Table 1. For each file, we specify: (*i*) a brief description of the file content; (*ii*) the format of the file; (*iii*) a flag that indicates if the file is mandatory; (*iv*) the default values used by FiCoS, when applicable. Notice that all indices are 0-based and the files are in Tab-Separated Value (TSV) format. In the table, N is the number of chemical species, M the number of reactions involved in the model, and E the number of required simulations. For each simulation, T represents the number of sampling time instants in which the dynamics must be saved. FiCoS can accept a single parameterization consisting in the initial amounts of all species (either `M_0` or `MX_0`) and all kinetic constants (either `c_vector` or `c_matrix`), as well as multiple parameterizations described by a set of the initial amount of the species (`MX_0`) and a set of kinetic constants (`c_matrix`). Finally, FiCoS allows for computing in parallel a difference between the obtained simulations and a target series (`c=ts_matrix`). Specifically, FiCoS implements the fitness function described in [1].

References

[1] Andrea Tangherloni, Simone Spolaor, Paolo Cazzaniga, Daniela Besozzi, Leonardo Rundo, Giancarlo Mauri, and Marco S Nobile. Biochemical parameter estimation vs. benchmark functions: a comparative study of optimization performance and representation design. *Appl. Soft Comput.*, 81:105494, 2019.

Table 1: FiCoS input files

File name	*Content*	*Format*	*Optional*	*Default*
`alphabet`	Vector containing the chemical species names	N columns	Yes	X_j, with j=0, . . . , N-1
`left_side`	Stoichiometric matrix of the reactants[1]	M rows, N columns	No	
`right_side`	Stoichiometric matrix of the products[2]	M rows, N columns	No	
`c_vector`	Vector of kinetic constants[3].	M rows	Yes, if `c_matrix` is provided	
`c_matrix`	Matrix of kinetic parameters[3]	E rows, M columns	Yes, if `c_vector` is provided	
`M_0`	Vector of the initial amounts of chemical species[4]	N columns	Yes, if `MX_0` is provided	
`MX_0`	Matrix of the initial amounts of chemical species[4]	E rows, N columns	Yes, if `M_0` is provided	
`t_vector`	Vector of sampling time instants	T rows	No	
`M_feed`	Matrix of the chemical species whose values must be kept constant[5]	N columns or<br>E rows, N columns	Yes	$\mathbf{0}$
`modelkind`	Type of input model[6]	{stochastic, deterministic}	Yes	deterministic
`volume`	Reaction volume of the system[7]	real number	Yes	
`cs_vector`	Vector of chemical species to be saved	$K \leq N$ rows	Yes	All chemical species
`atol_vector`	Vector of absolute error tolerances	N rows	Yes	$\mathbf{10^{-12}}$
`rtol`	Relative tolerance	real number	Yes	10^{-6}
`max_steps`	Maximum number of allowed integration steps	real number	Yes	10000
`ts_matrix`	Matrix of target time series. The first column must be equal to `t_vector`.	T rows, $K+1$ columns (with $K \leq N$)	Yes	

[1]Left-hand side of the reactions.
[2]Right-hand side of the reactions.
[3]Both deterministic or stochastic kinetic constant values, associated with the reactions, are allowed (see `modelkind` file).
[4]Both concentration (real numbers) or molecular amount (integer number) values are allowed (see `modelkind` file).
[5]This vector/matrix indicates the chemical species whose amounts must be kept constant throughout the simulation (i.e., they are assumed to be constantly fed into the system). The values in this vector/matrix are equal to 0 if the species can vary in time, they are equal to 1 otherwise (keeping the values equal to the related values in `M_0` or `MX_0`).
[6]Deterministic: amount of chemical species given as concentration values, reaction parameters specified as deterministic reaction rates. Stochastic: amount of chemical species given as integer numbers of molecules, reaction parameters specified as stochastic constants.
[7]Required if the model is defined as stochastic in the `modelkind` file. It is exploited to convert the stochastic kinetic constants into the deterministic formulation as well as the molecular amounts (integer number) into concentrations (real numbers).
