# Supplementary material for: FiCoS: A fine-grained and coarse-grained GPU-powered deterministic simulator for biochemical networks
Source: PLoS Comput Biol. 2021 Sep 9;17(9):e1009410. doi: 10.1371/journal.pcbi.1009410 (PMC8476010; doi:10.1371/journal.pcbi.1009410)
Supplement: Attachment — Submitted filename: ResponseReviewers.pdf [file pcbi.1009410.s003.pdf]

Dear Dr. Dina Schneidman,

We would like to thank you for your consideration of our manuscript "*FiCoS: a fine- and coarse-grained GPU-powered deterministic simulator for biochemical networks*" (**PCOMPBIOL-D-21-00064**).

We would also like to thank the Reviewers for their comments, which allowed us to further improve our manuscript as well as our simulator. We made the source code publicly available, along with relevant tutorials and examples, at the following GitLab repository: https://gitlab.com/andrea-tango/ficos.

Please find below our point-by-point response to all the comments raised by the Reviewers. All changes implemented in the manuscript have been highlighted in red colour.

We hope that, with these improvements, you will find our manuscript suitable for publication in the PLoS Computational Biology journal.

Yours sincerely,
Andrea Tangherloni, on behalf of all co-authors

PLoS Computational Biology Bioinformatics: PCOMPBIOL-D-21-00064
Responses to the Reviewers' Comments (Round 1)

Reviewer #1

Comment 1. I could not find any mention of where to get the software FiCoS, Without that I cannot review the paper properly. Even a search on google did not bring up any reference to the software.

Authors. The authors would like to thank the Reviewer for his/her feedback. We made the source code publicly available, along with relevant tutorials and examples, at the following GitLab repository: https://gitlab.com/andrea-tango/ficos.
Specifically, we uploaded the source code of both the CPU and GPU versions of FiCoS, all the scripts used to generate the results presented in the manuscript, and two Jupyter Notebooks. In these Jupyter Notebooks, we provided all the necessary information to run a 2-Dimensional Parameter Sweep Analysis and a Sensitivity Analysis exploiting FiCoS. Finally, we listed and explained all the input parameters of FiCoS. We hope that now both novice and expert researchers can successfully run parallel simulations of Reaction-based Models, by relying on the computational power of FiCoS.

Reviewer #2

This paper presents a novel approach for conducting fine- and coarse-grain parallel simulations of ODE systems arising from biochemical reaction networks. The introduction provides a fairly comprehensive overview of previous efforts in this area and the body of the paper presents results of a series of comparisons between the new method and previous ones, including the widely-used standard single processor CPU-based implementations. Most of the comparisons use synthetic networks, but the final application is a case study on a large-scale existing model of autophagy, which demonstrates that the FiCoS approach is capable of performing a much larger number of ODE integrations given a fixed time budget. The method is described schematically in the Methods section at the end of the paper and in much greater detail in Supplemental material. Overall, this division of material seems appropriate. The paper is generally well written and easy to follow.

Authors. The authors would like to thank the Reviewer for his/her useful suggestions.

Comment 1. I have two main concerns about the paper. First, I believe this is intended to be an open source freely available tool (e.g., line 232 refers to the tool as "open source") but this is not made clear in the text and no links to the current implementation are provided. I feel that the significance of this work for the computational biology community would be greatly diminished if the tool and code were not available. I am sure that this point is easily addressed.

Authors. We made the source code of both the CPU and GPU versions of FiCoS freely available for download at the following GitLab link: https://gitlab.com/andrea-tango/ficos.
We also uploaded all the scripts used to generate the results presented in the manuscript.

Comment 2. The second concerns demonstration of the practical relevance of the tool. GPU's offer the potential to greatly increase the scale of computational analysis that can be performed on biological models, and the case study presented highlights how the method can be applied to a parameter sweep analysis, which is a commonly-performed task. However, I think it would strengthen the impact and interest of this paper to present demonstrations of one or two other common analyses, such as parameter sensitivity and parameter estimation, and to demonstrate how GPU-based simulations can be used in such practical applications to obtain superior performance. I would strongly encourage the authors to present their case studies in the form of Jupyter notebooks that would be available for the community to adapt for use in their own applications. I think a few examples like this could greatly increase the adoption of GPU use in the field compared to the current state, where I think most practitioners are not using this technology.

Authors. We have prepared and shared in the GitLab repository two Jupyter Notebooks to show how FiCoS can be used to speed up practical applications of Computational and Systems Biology. Specifically, we created a Jupyter Notebook to run a 2-Dimensional Parameter Sweep Analysis (PSA), and a Jupyter Notebook to run a Sensitivity Analysis (SA), exploiting the computational power of FiCoS. Specifically, the PSA Jupyter Notebook shows the analysis of the model of the Autophagy/Translation switch based on the mutual inhibition of MTORC1 and ULK1 [Szymańska P. *et al.*: *"Computational analysis of an autophagy/translation switch based on mutual inhibition of MTORC1 and ULK1"*, PLoS One, 10(3), e0116550, 2015], while in the SA Jupyter Notebook we analysed the impact of the effects of an isoform-specific modification on the system outcome of a model of the human intracellular metabolic pathway in red blood cells [Totis N. *et al.*: *“Efficient and settings-free calibration of detailed kinetic metabolic models with enzyme isoforms characterization”*, proc. of International Meeting on Computational Intelligence Methods for Bioinformatics and Biostatistics, p. 187–202, Springer, 2018].
We believe that these examples will help strengthen the impact and interest that FiCoS might bring to the scientific community. These examples can be easily adapted to tackle similar problems by researchers with different backgrounds and working in different fields. We also hope that FiCoS will allow for substantially increasing the adoption of GPU-based simulators, a solution that can drastically speed up the analyses of Computational and Systems Biology.
