# Supplementary material for: FiCoS: A fine-grained and coarse-grained GPU-powered deterministic simulator for biochemical networks
Source: PLoS Comput Biol. 2021 Sep 9;17(9):e1009410. doi: 10.1371/journal.pcbi.1009410 (PMC8476010; doi:10.1371/journal.pcbi.1009410)
Supplement: Attachment — Submitted filename: ResponseReviewers_R2.pdf [file pcbi.1009410.s004.pdf]

Dear Dr. Dina Schneidman,

We would like to thank you for your consideration of our manuscript "*FiCoS: a fine- and coarse-grained GPU-powered deterministic simulator for biochemical networks*" (**PCOMPBIOL-D-21-00064R1**).

We would also like to thank Reviewer 1 for his/her suggestions, which allowed us to further improve our manuscript.

Please find below our point-by-point response to all the comments raised by the Reviewer. As required, all changes implemented in the manuscript have been highlighted in red colour.

We hope that, with these modifications, you will find our manuscript suitable for publication in the PLoS Computational Biology journal.

Yours sincerely,
Andrea Tangherloni, on behalf of all co-authors

PLoS Computational Biology Bioinformatics: PCOMPBIOL-D-21-00064R1
Responses to the Reviewer' Comments (Round 2)

Reviewer #1

Comment 1. This is an interesting paper on speeding up mass-action based chemical models using GPUs. In fact this is the first paper I've seen where I am actually impressed with the performance. There are limitations, in particular the approach only deals with mass-action kinetics and as far as I can tell it cannot deal with nonlinear rate laws such as Hill of Michaelis-Menten like expressions. Do the authors have any plans to extend the applicably to more complex rate laws? A mention of this could be made in the discussion, eg what would be the issue to implement such capabilities?

Authors. We would like to thank the Reviewer for his/her feedback, and we are glad to hear that the manuscript was positively received by him/her. We agree with the Reviewer that dealing only with mass-action kinetics can be a limitation; thus, as explained in the "Conclusions" section, we will extend FiCoS to take into account additional kinetic rate laws.

This current limitation arises from the way ODEs are encoded by FiCoS and are parsed by the CUDA kernels. We are planning an improved version of this encoding, able to represent Hill and Michaelis-Menten kinetics, in addition to mass-action kinetics. However, we expect that the new parser will slightly affect the performance of FiCoS, mainly because of an increased level of conditional branching and higher latencies due to additional global memory accesses. In addition, we are evaluating the possibility of defining a general-purpose version of FiCoS, capable of simulating models whose reactions follow arbitrary kinetics. In this case, the main issue would concern the complexity of encoding arbitrary equations and the resulting difficulty of calculating partial derivatives for the Jacobian matrix. A possible solution would be meta-programming, following a strategy similar to ginSODA, in which the kernels are partially rebuilt and dynamically linked at run-time. In any case, it would be necessary to adapt this approach to FiCoS, being unfeasible for fine-grained parallelization.

Comment 2. The real bottleneck today in computational systems biology is parameter fitting where most of the time is spend in solving the ODEs. In our own work it can take 36 hours to fit a single model on a 8 core machine and we have many 1000s to fit. The second reviewer also mentioned this problem but it doesn't appear to have been addressed by the reviewers. The authors might not be able to solve this problem at the moment but there should be some comment on this is the discussion section and whether a GPU approach could solve this.

Authors. FiCoS can be efficiently coupled with any optimization algorithm to efficiently perform a Parameter Estimation (PE). We added to Section "Human intracellular metabolic pathway" the results concerning the PE of the RBM of the human intracellular metabolic pathway in red blood cells. Specifically, we coupled FiCoS with a setting-free version of Particle Swarm Optimization, named Fuzzy Self-Tuning PSO, to estimate the 78 missing kinetic constants of this RBM. According to our tests, FiCoS was able to execute the PE around 30 times faster than LSODA.

Comment 3. In terms of comparison it might have been better to compare against CVODE rather than LSODA. CVODE is a much more modern integrator, LSODA is now over 25 years old is unchanged during that time.

Authors. We agree with the reviewer, indeed, the performance of FiCoS was compared against both LSODA and VODE, which are the most used ODE solvers. Specifically, we exploited the versions implemented in the Python SciPy library, which provides wrappers to the solvers implemented in the ODEPACK, a collection of efficient Fortran solvers. CVODE, the solver suggested by the Reviewer, is a porting in C programming language of the Fortran packages VODE and VODPK [Cohen S.D. *et al.*: *"CVODE, a stiff/nonstiff ODE solver in C"*, Computers in Physics, 10(2), p. 138-143, 1996] included in ODEPACK. Therefore, the tests presented in the manuscript already include a comparison against the integration method suggested by the reviewer.

Comment 4. No mention of SBML is made at all in the paper or supplement. Most models are now stored in SBML (see biomodels), how are users to exploit the new GPU code given that models are in SBML? As a result no comparisons were made with existing simulators such as COAPSI, VCell, roadrunner etc.

Authors. We included in the GitLab repository a tool to convert SBML models into the BioSimWare format [Besozzi D. *et al.*: *"BioSimWare: A Software for the Modeling, Simulation and Analysis of Biological Systems"*, proc. of International Conference on Membrane Computing, p. 119-143, Springer, 2010], currently used by FiCoS, in addition to LASSIE and cupSODA simulators. We are currently working on an improved version of this tool to convert a BioSimWare model into the corresponding SBML, taking also into account all model parameterizations that can be defined to run a Systems Biology analysis (e.g., Sensitivity Analysis, Parameter Estimation, Parameter Sweep Analysis).
Regarding the comparisons, we directly evaluated the performance of FiCoS against vanilla ODE solvers to achieve a fair comparison among them. Indeed, we were able to separately measure the time required by the integration methods and the time spent by the simulators for I/O operations. In addition, we would like to point out that the tools mentioned by the Reviewer exploit the ODE solvers taken into account in our tests. To be more precise, libRoadRunner uses CVODE and a standard fourth-order Runge–Kutta method; COPASI performs deterministic simulations using LSODA and an implementation of the RADAU5 method; VCell exploits CVODE and other simpler integration algorithms.

Comment 5. I was very happy to see that code is now open source. Note that the use of GPL-3 will likely significantly restrict it use by the community., perhaps a more liberal open license would be better? Of course the authors have the final say in the type of license they use.

Authors. We agree with the Reviewer that the GPL-3 is quite restrictive; therefore, we decided to opt for the BSD 3-Clause License. We modified both the manuscript and repository accordingly.

Comment 6. Finally, the supplement mentioned the hardware that was used, "All tests were performed on a workstation equipped with a CPU Intel Core i7-2600 CPU (clock 3.4 GHz) and 8 GB of RAM, running on Ubuntu 16.04 LTS. The GPU used in the tests was a Nvidia GeForce GTX Titan X (3072 cores, clock 1.075 GHz, RAM 12 GB), CUDA toolkit version 8 (driver 387.26)." I would move this to the methods section in the main text because if someone want to use this code, they need to know what hardware to purchase and the method section the best place for this. For example this reviewer is interested in trying out the code.

Authors. We completely agree with the Reviewer that the information about the hardware used in the tests is fundamental for readers interested in using FiCoS. Therefore, this information was already reported both in the supplementary file and in the Results section of the main text (line 119 of the manuscript).
